# The association between the levels of burnout and quality of life among fourth-year medical students at the University of the Free State

**DOI:** 10.4102/sajpsychiatry.v24i0.1101

**Published:** 2018-07-25

**Authors:** Lauren Colby, Moliehi Mareka, She’neze Pillay, Fatima Sallie, Christine van Staden, Edwin D. du Plessis, Gina Joubert

**Affiliations:** 1 School of Medicine, University of the Free State, South Africa; 2 Department of Psychiatry, University of the Free State, South Africa; 3 Department of Biostatistics (G31), University of the Free State, South Africa

## Abstract

**Background:**

Society invests huge financial resources in training medical students. However, the academic and personal demands placed on these students can be taxing and may be detrimental to students’ quality of life leading to high levels of burnout and academic dropout rates.

**Aim:**

To determine the association between the levels of burnout and quality of life among fourth-year medical students at the University of the Free State (UFS).

**Setting:**

School of Medicine, UFS, Bloemfontein.

**Methods:**

All fourth-year medical students in their first semester of the clinical phase were included. Data were collected using anonymous self-report measures. The Maslach Burnout Inventory (MBI) measured the levels of burnout according to three subscales (emotional exhaustion, depersonalisation and personal achievement), and the World Health Organization Quality of Life Assessment (WHOQOL-BREF) measured the quality of life.

**Results:**

Of the 121 enrolled fourth-year medical students, 91 (75.2%) completed the questionnaires. The MBI personal achievement subscale had the highest number of participants (*n* = 53; 58.2%) with high levels of reported burnout. Significant associations were found between the psychological health subscale of the WHOQOL-BREF and all three subscales of the MBI, in particular emotional exhaustion.

**Conclusion:**

An association exists between the levels of burnout and quality of life among fourth-year medical students. This information could be of value to medical schools as they are in a position to implement interventions that promote students’ well-being.

## Introduction

Medical students are faced with gruelling academic demands on the one hand and personal demands on the other. These demands can be very taxing and may be detrimental to students’ quality of life.^1^ Factors that negatively affect students’ quality of life are linked to the academic environment and an inability to manage time well.^2^ The transition to the clinical phase during the undergraduate medical programme presents another occasion for intense anxiety, uncertainty and fear caused by feelings of limited scientific knowledge. Heavy workload and educational content, combined with high levels of educational demands, a lack of leisure time and limited contact with family and friends are other factors that lessen the quality of life.^3,4^ A poor quality of life coupled with burnout is likely to jeopardise students’ ability to reach the most optimum levels of training and have implications for their future competencies as clinicians.^5^

Factors associated with the teaching aspects of the medical programme that can improve the quality of life include good lecturers, didactically taught classes, well-designed assessments and shorter duty hours during clinical years. These factors were also reported to decrease burnout rates among medical residents.^2,6^ Participating in various social activities increases quality of life, as it improves self-esteem and usefulness when the students carry out their social role.^2^ Quality of life is further improved by maintaining meaningful personal relationships.^2^

Burnout describes a state or process of mental exhaustion.^7^ More specifically, burnout refers to a multi-dimensional construct consisting of emotional exhaustion, cynicism (or depersonalisation) and professional inefficacy.^8,9^ Burnout has been shown to have a devastating impact on health professionals, such as declining mental and physical health as well as quality of life, which has serious repercussions for health professions as the career becomes less attractive. According to literature, distress in medical students can lead to burnout with significant consequences.^10^ Compared to the general population, medical students and medical practitioners have a higher prevalence of burnout and stress-related mental disorders.^11^

The experience of a poor quality of life, high levels of burnout and stress are contributing factors that adversely affect the development of students’ knowledge, skills and professionalism, and can have profound consequences, such as suicide ideation and intent to drop out of medical school.^12^

### Aim

The aim of this study was to determine the association between the levels of burnout and quality of life among the fourth-year medical students at the University of the Free State (UFS).

## Research methods and design

### Study design

This was an analytical, cross-sectional study design.

### Setting

School of Medicine, UFS.

### Study population and sampling strategy

The study population included all enrolled fourth-year medical students in semester 6 (the first semester of the clinical phase of the medical programme) at the UFS during 2015. This comprised 121 students of which 63 were enrolled in the English class and 58 in the Afrikaans class.

Students who were not present on the day of the survey were excluded from the study. There was no intervention.

### Data collection

Data were collected using anonymous self-report measures consisting of three parts: (1) questions on the socio-demographic characteristics of the sample (age, gender, race, home language, marital status, highest qualification and current residence), (2) the Maslach Burnout Inventory (MBI) questionnaire and (3) the World Health Organization Quality of Life Assessment (WHOQOL-BREF) survey. Questionnaires were available only in English and were distributed to both the English and Afrikaans classes after their respective lectures for immediate completion.

### Measuring instruments

Burnout was measured with the Maslach Burnout Inventory – Human Services Survey (MBI-HSS).^8^ The MBI assesses various aspects of burnout in professionals from different sectors. Psychometric analyses have shown that the scale is a highly reliable and valid measure of burnout.^13,14^ The MBI-HSS consists of 22 items^14^ which are scored on a seven-point frequency rating scale ranging from 0 (never) to 6 (daily).^15^ The risk of burnout is explored by assessing three components: emotional exhaustion (EE), depersonalisation (DP) and personal achievement (PA).^16^ The EE scale measures feelings of being emotionally overextended and exhausted by one’s work. The DP scale measures an impersonal response towards care treatment or instruction, and the PA scale measures feelings of competence and success.^17^ High scores in EE and DP and low scores in PA are indicative of burnout.^15^

The extent of burnout is determined by the combination of levels of burnout for each subscale. The MBI guidelines^8^ define the levels of burnout as low (low levels in all three subscales), moderate (moderate levels in all three subscales) and high (high levels in all three subscales). As there are more possible combinations than listed in the MBI guidelines,^8^ the researchers decided to interpret the data as follows:

- **Low:** three subscales ‘low’, or two subscales ‘low’ and one subscale ‘moderate’
- **Moderate:** three subscales ‘moderate’, or two subscales ‘moderate’ and one subscale ‘low’ or ‘high’
- **High:** three subscales ‘high’, or two subscales ‘high’ and one subscale ‘moderate’.

Quality of life was measured with the WHOQOL-BREF survey,^18^ an international cross-culturally comparable assessment available in different languages for both developed and developing countries.^19^ The WHOQOL-BREF^18^ consists of 26 questions ranked according to a five-point Likert scale and measures four domains: physical health, psychological health, social relations and environment (collectively referred to as the QoL domains). Points for each domain are linearly changed to a score from 0 to 100. Higher scores out of the 100 are indicative of a better quality of life.^20^

### Pilot study

A pilot study was conducted at the Faculty of Health Sciences, UFS, on five randomly selected third-year (Semester 4) medical students: four students from the English class and one student from the Afrikaans class. They were provided with an information leaflet and asked to complete the 3-part questionnaire. The results obtained from the pilot study were not included in the data analysis.

A few introductory questions from the WHOQOL-BREF survey were repeated in the biographical questionnaire. These included age, gender and ‘are you currently ill?’ Thus, these questions were removed from the final biographical questionnaire as no changes to the set questions of the WHOQOL-BREF are allowed.

### Data analysis

Data were analysed by the Department of Biostatistics, Faculty of Health Sciences, UFS. The results were summarised as frequencies and percentages (categorical variables), and medians and percentiles (numerical variables). Intercorre-lations between the variables were determined with the Spearman’s rank correlation coefficient. Subgroup comparisons were performed using the Kruskall-Wallis test.

## Ethical consideration

The study was approved by the Ethics Committee of the Faculty of Health Sciences, UFS. Permission to collect and analyse the data was obtained from the Vice-rector: Research, Dean of the Faculty of Health Sciences, Dean of Student Affairs and Head of the School of Medicine, UFS. The MBI was purchased from Mind Garden, Inc. Permission was obtained from the WHO to use the WHOQOL-BREF. Participants were informed about the voluntary nature of their participations, as well as the anonymity and confidentiality of individual responses. An information leaflet, in both languages, was also distributed to the students, informing them of available psychological services on campus.

## Results

Of the 121 fourth-year medical students, 91 completed the questionnaires (response rate 75.2%). Participants were mostly female (57.1%), white people (81.1%) and between the ages of 19 and 32 years (median 21 years). The predominant home language was Afrikaans (60.4%). Most of the participants were single (86.8%), and 79.1% of the participants lived off campus.

### Levels of burnout in students

An equal number of participants had either low (*n* = 36; 39.6%) or high (*n* = 36, 39.6%) levels of burnout in the EE subscale (Table 1). For the DP subscale, the largest percentage had moderate levels of burnout (*n* = 38, 41.7%). The PA subscale had the highest number of participants (*n* = 53; 58.2%) with high levels of burnout out of all three subscales.

**TABLE 1:** Summary of the Maslach Burnout Inventory burnout subscales and the quality of life domains.

Variable	Results					
**MBI subscales**	**Low** *n* **(%)**		**Moderate** *n* **(%)**		**High** *n* **(%)**	
Emotional exhaustion (EE)	36	(39.6)	19	(20.9)	36	(39.6)
Depersonalisation (DP)	27	(29.7)	38	(41.7)	26	(28.6)
Personal accomplishment (PA)	11	(12.1)	27	(29.7)	53	(58.2)
**QoL domains**	***n*†**	**median (IQR)**				
Physical health	91	69 (63–81)				
Psychological health	89	69 (50–75)				
Social relationships	78	69 (50–81)				
Environment	90	75 (69–88)				

† , Where *n* differs from the group sample size, it is because of items being omitted on the questionnaire and a score not being calculated.

IQR, interquartile range; MBI, Maslach Burnout Inventory; QoL, quality of life.

In total, 65 participants were categorised according to the pre-selected combinations (as mentioned in the Methods section). Most of these participants (*n* = 30; 46.1%) showed high levels of burnout, 22 (33.8%) showed moderate levels of burnout and 13 (20.0%) showed low levels of burnout. The 26 participants who were not categorised consisted mainly of students who had low on one subscale, moderate on another and high on the third. According to the MBI-prescribed categorisation, only 20 students would have been classified: 4 (20.0%) low levels of burnout, 2 (10.0%) moderate levels and 14 (70.0%) high levels.

### Quality of life

Environment had the highest median of 75 (on the scale from 0 to 100), whereas all other QoL domains had a median of 69 (Table 1).

### Levels of burnout and quality of life

Table 2 shows that burnout categories of EE and DP differed significantly regarding scores in all four QoL domains, with psychological health being the most significant. There is generally a clear trend of decreasing medians on QoL domains between the MBI subscales low, moderate and high. For the PA subscale, significant differences were only found for psychological health. Spearman’s rank correlations indicated that all domains had the highest correlations with the EE subscale scores with correlations ranging from -0.68 (psychological health) to -0.39 (environment).

**TABLE 2:** Median scores of the quality of life domains in the Maslach Burnout Inventory subscale categories.

QoL domains	MBI subscales						*p*
	Low		Moderate		High		
	*n*†	median (IQR)	*n*†	median (IQR)	*n*†	median (IQR)
**Emotional exhaustion (EE)**							
Physical health	36	81 (69–88)	19	75 (63–81)	36	63 (50–69)	< 0.01
Psychological health	35	75 (69–81)	19	69 (63–75)	35	50 (38–69)	< 0.01
Social relationships	30	78 (69–94)	17	69 (50–75)	31	56 (31–69)	< 0.01
Environment	36	88 (75–91)	18	75 (69–81)	36	69 (56–75)	< 0.01
**Depersonalisation (DP)**							
Physical health	27	81 (69–88)	38	72 (63–81)	26	63 (56–75)	< 0.01
Psychological health	26	75 (69–81)	37	69 (63–81)	26	50 (44–69)	< 0.01
Social relationships	24	75 (69–94)	30	72 (56–81)	24	56 (47–69)	0.006
Environment	27	81 (69–94)	37	75 (69–88)	26	69 (63–81)	0.037
**Personal accomplishment (PA)**							
Physical health	11	81 (63–88)	27	75 (63–88)	53	69 (63–81)	0.161
Psychological health	10	78 (69–88)	27	69 (63–81)	52	63 (44–75)	0.005
Social relationships	9	69 (69–81)	22	69 (56–94)	47	69 (50–75)	0.620
Environment	10	72 (69–81)	27	75 (69–94)	53	75 (63–88)	0.157

† , Where *n* differs from the group sample size, it is because of items being omitted on the questionnaire and a score not being calculated.

IQR, interquartile range; MBI, Maslach Burnout Inventory; QoL, quality of life.

A significant difference (*p* < 0.001) was noted for the physical health and psychological health QoL domains between the three overall burnout categories (Table 3). The same trend of decreasing medians on QoL domains as was observed on the individual burnout subscales is also observed here between overall low, moderate and high.

**TABLE 3:** Median scores of the quality of life domains in the Maslach Burnout Inventory overall categories.

QoL domains	MBI overall levels of burnout†						
	Low (*n* = 13)		Moderate (*n* = 22)		High (*n* = 30)		*p*
	*n*	median (IQR)	*n*	median (IQR)	*n*	median (IQR)	
Physical health	13	88 (81–94)	22	75 (69–81)	30	63 (50–69)	< 0.001
Psychological health	12	84 (72–94)	22	69 (63–81)	30	50 (38–69)	< 0.001
Social relationships	11	81 (69–100)	17	69 (56–81)	27	56 (31–75)	0.005
Environment	13	88 (81–94)	21	75 (75–88)	30	69 (56–81)	0.001

† , ***n*** = 26 excluded because of not fitting into overall low, moderate or high categories.

IQR, interquartile range; MBI, Maslach Burnout Inventory; QoL, quality of life.

## Discussion

A Brazilian study that assessed the association between empathy, quality of life and burnout in medical students found the presence of burnout in all stages of medical education with students in their final year scoring higher in EE and DP.^21^ In our study of fourth-year students, PA was the most common burnout dimension in the high category. Median scores on the QoL domains were found to be fairly high, thus indicative of good quality of life.

The results of the MBI subscales indicate that one’s physical health, psychological well-being, social relationships and environment are all associated with levels of burnout. White^22^ stated that ‘emotional exhaustion occurs when you have exceeded your capacity for emotional stress’. She also noted that EE results in physical symptoms and a feeling of being psychologically and emotionally drained. The results of our study showed a stronger association between students’ psychological health and EE.

Pagnin and Queiroz^23^ investigated the influence of burnout and sleep difficulties on the quality of life of medical students. In their study, the association between the EE subscale and the physical health domain showed the greatest significance (*p* = 0.001), but they highlighted that both the physical health and psychological health domains are affected by burnout and sleeping difficulties. They also noted the negative toll poor psychological health can inflict on the quality of life of medical students.

An equal number of students scored either low or high in the EE subscale. This suggests that, although many fourth-year medical students are emotionally exhausted, there are also as many students who are not. In comparison, Pagnin and Queiroz^23^ found that two-thirds of medical students felt emotionally exhausted at least once a week.

Depersonalisation and psychological health showed a significant association in this study. Depersonalised students may experience feelings of cynicism and detachment towards patients and often engage in unprofessional behaviour.^21^ Negative feelings, low self-esteem and disbeliefs in the psychological health domain can be linked to a detached attitude.^23^

A possible explanation for the students scoring moderate and high in DP and low in psychological health could be the result of a high academic overload because of clinical rotations and closer contact with patients.^21^

A decrease in PA means that an individual assesses himself or herself negatively and is unable to move forward in the situation. The person begins to doubt his or her genuine abilities to accomplish things. This domain may also represent demotivating effects of a difficult, repetitive situation leading to failure, despite many efforts.^16^

As found in our study, there was a significant association between PA and psychological health among fourth-year medical students. Paro et al.^21^ demonstrated significant associations between PA and psychological quality of life and diminished personal distress among students. They also reported that PA and professional growth are linked with empathic dispositions among adults and higher satisfaction with care among patients.

Henning et al.^24^ reported QoL results of medical students in Auckland, New Zealand, and compared these to results of non-medical students at other universities in the same city and an Australian population norm. The students groups had similar results, but lower values than the population norm. Optimising all students’ quality of life should thus be a priority for educational institutions. Medical educators, in particular, should give attention to this because recent findings suggest that perceptions of emotional, psychological and social well-being are positively associated with students’ altruistic professional values.^21^

### Study limitations

Although this study provided noteworthy findings, the results should be interpreted with care, especially as far as their generalisation is concerned. Only fourth-year medical students from the UFS were included in the study, and thus, the sample is also small. This was a cross-sectional study where data were gathered at one specific point in time, and thus, the direction of the association cannot be determined. Furthermore, it is possible that the results may have been influenced by circumstances at the time the questionnaires were completed. The study was conducted before a semester test and some students did not attend classes in order to study. Students were aware that the questionnaire dealt with burnout and quality of life, and could thus have been sensitised, but the rating of items was not available to them. Other factors that may impact on both QoL and burnout and their interrelationship (e.g. resilience and social support) were not assessed. The results obtained may also not translate to medical students in different academic years, or fourth-year students at other South African medical schools.

The questionnaire was only made available in English, and as most of the participants were not English first-language speakers, they may have misunderstood some of the questions or instructions. Improvements could have been made to the response rate by asking the lecturer in advance to have a time before class, instead of after class as we have done, to conduct the study.

The MBI does not specify categories for overall levels of burnout, and the researchers consequently devised their own categorisation. These categories have not been validated. A WHOQOL domain score could not be calculated if any of the items was coded ‘missing’. This implies that a few participants had to be excluded from the total number of observations in a domain.

## Conclusion

Fourth-year medical students’ physical health, psychological well-being, social relationships and environment are all related to their levels of burnout. This information could be of value to medical schools as they are in a position to implement possible interventions that could promote students’ well-being. Interventions, such as stress management, have been shown to prevent burnout by reducing stress and possibly improving quality of life.^25^

### Recommendations

Medical schools can promote student’s well-being through teaching and promoting self-care skills, instituting wellness interventions and educating students about preventing and reducing burnout.^25,26^ The restructuring of medical student duties, regular performance evaluations and mentoring programmes have all been reported to be helpful in reducing medical student burnout.^27^ It is proposed that interventions such as counselling, instrumental support, encouragement of physical exercise and stress management courses be part of the curriculum at medical schools.
